# Missed Opportunities in Clinical Psychology: What About Running Factorial Design Internet Trials and Using Other Outcomes Than Self-Report?

**DOI:** 10.32872/cpe.12063

**Published:** 2023-06-29

**Authors:** Gerhard Andersson

**Affiliations:** 1 Department of Behavioural Sciences and Learning and Department of Biomedical and Clinical Sciences, Linköping University, Linköping, Sweden; 2 Department of Clinical Neuroscience, Karolinska Institutet, Stockholm, Sweden

Clinical psychology and in particular research on and implementation of psychological treatments can be regarded as a success story (Hofmann et al., 2012). Many treatment guidelines and recommendations now acknowledge that psychological treatments can serve as adjuncts to pharmacological treatments, and they are also described as stand-alone and first-line recommended treatments for mild to moderate psychological problems and diagnoses like major depression and the anxiety disorders. The reason for this is not based on opinion and consensus (which used to be the case in medicine and psychiatry 100 years ago), but increasingly well conducted research studies inform health care and the practice of clinical psychology. Not only controlled intervention studies change practice but also research on mechanisms and processes including self-report measures, brain-imaging and tests of information processing, to give a few examples. In particular, when it comes to cognitive-behavioural treatments (CBT), it can rightfully be argued that there is less need for new studies repeating the same finding that getting CBT is often better than not getting it (there might still be a need to study different psychotherapy orientations like psychodynamic psychotherapy). One way to bring intervention research forward is to use factorial designs in order to discern effective components (Watkins & Newbold, 2020). As I will return to it has not been possible to obtain large enough sample sizes in regular clinical research to run factorial design trials but the use of the internet and modern information technology has changed this (Andersson et al., 2019).

## Are There Any Problems?

But there are problems. Being an intervention researcher having done many controlled trials I am aware of the fact that almost all outcome studies in clinical psychology ONLY rely on self-report measures. These are relevant, valid, and sensitive to change and should not be removed from research. A treatment study on say major depression should definitely include a validated measure of symptoms of depression (like for example the Beck Depression Inventory). Trials also benefit from adding measures of other constructs like quality of life, health care consumption and sometimes also repeated administration of self-report measures to capture change processes and study mediation. However, what happened to actual behaviour? In my PhD I had a trial on older adults with hearing loss including a behavioural test of communication skills (Andersson et al., 1995). Later when we began doing trials on the internet we included a behavioural approach test in studies on specific phobia (e.g., Andersson et al., 2013). More recently I was part of a trial on virtual reality exposure for spider phobia using the standard behavioural approach task (Miloff et al., 2019). But with those and a few other exceptions most of the trials I have been involved with have not included any direct observation of behaviour. It is important to note the ecological momentary assessment (EMA) very often is just another format for self-report of behaviour. There are exceptions, for example sleep and activity monitoring, but overall modern information technology and smartphones have not been used often as ways to collect behavioural outcomes, in spite of calls for such research (Mohr et al., 2017).

## Modern Information Technology as a Way to Speed Up the Process

Clinical psychology and psychotherapy research overall has benefitted much from technological innovations and in particular computerized assessments and treatment delivery over the internet. Now internet intervention trials can be larger, less costly, reach more people and also suffer less from data loss compared to traditional studies (Schuster et al., 2021). As I mentioned it is now also possible to run factorial design trials with better power than used to be the case in traditional face-to-face studies. I will use an example of a factorial design trial in which we both measured and manipulated one crucial aspect of most psychological treatments namely knowledge and the role of learning support. We began studying knowledge acquisition more than 10 years back (Andersson et al., 2012), but returned to the topic and were also inspired by Harvey and co-workers (2014). In Berg et al. (2020) we included 120 adolescents who suffered from mixed anxiety/depression. They were randomised to one of four treatment groups, in a 2×2 design with two factors: with or without learning support and/or chat-sessions. We did not have a waitlist control group. Interestingly and in addition to large improvements overall we found that adding learning support (different ways to boost learning of treatment material) lead to larger effects on the Beck Anxiety Inventory (*d* = 0.38), and also increased knowledge gain (*d* = 0.42), when compared against the group who did not receive this boost of learning. To our surprise chat-sessions did not have any additional effects. The point here is that knowledge has not been the focus of much research in spite of the fact that in particular CBT focus on psychoeducation and that clients both understand and remember the rationale behind the treatment techniques. My second point is that internet intervention research can speed up our understanding of what works for whom and more rapidly test new ideas by for example adding behavioural outcomes.

## Future Hopes for Psychologists

I hope future research can inform us more about actual behavioural change including cognitive aspects of everyday function. There is so much more to do. To take one example, prospective cognition is something we use on a daily basis. Examples of prospective cognition can be for example to remember to take medication, call a friend or pick up milk at the grocery store when passing the dairy section in the store. Prospective cognition is most likely crucial for a client who has been in therapy when confronted with an unexpected trigger for anxiety (with avoidance being a likely reaction). The former client then needs to recall and practice what was learned and rehearsed in therapy (which can be years back). Surprisingly, this has not been studied much and we basically do not know how important it is for long term outcome following therapy.

In conclusion, I hope we can move our field forward by having larger samples, using factorial design and focus more on outcomes that have either been forgotten (behavioural change) or not even studied much (prospective cognition and knowledge).
